# Genes related to allergen exposure in allergic rhinitis: a gene-chip-based study in a mouse model

**DOI:** 10.1186/s12920-022-01389-4

**Published:** 2022-11-25

**Authors:** Min Wang, Ying Li, Jun Yang, Xiangdong Wang, Luo Zhang

**Affiliations:** 1 Department of Otolaryngology Head and Neck Surgery, Beijing Tongren Hospital, Capital Medical University, Beijing, 100730 China; 2 Beijing Laboratory of Allergic Diseases, Beijing Key Laboratory of Nasal Diseases, Beijing Institute of Otolaryngology, No 17, Hougouhutong, Dongcheng District, Beijing, 100005 China; 3 Department of Allergy, Beijing TongRen Hospitalm, Capital Medical University, Beijing, 100730 China; 4 Research Unit of Diagnosis and Treatment of Chronic Nasal Diseases, Chinese Academy of Medical Sciences, Beijing, 100730 China

**Keywords:** Allergic rhinitis, Mouse model, Allergen exposure, Microarray

## Abstract

**Background:**

The typical clinical symptoms of allergic rhinitis (AR) are known to be associated with allergen exposure; however, the underlying mechanisms are not fully understood. We wanted to gain a comprehensive view of the molecular mechanisms related to allergen exposure in a well-controlled mouse model of AR.

**Methods:**

An OVA-induced AR model was developed. Two hours and 4 weeks after the last OVA challenge, AR symptoms and local immune responses were assessed. At the same time, differentially expressed genes (DEG) in nasal mucosa were identified by gene expression microarray and further analyzed by bioinformatics methods. Verification of DEG was done by quantitative RT-PCR and immunohistochemistry.

**Results:**

The number of nasal rubbings and sneezes, serum OVA-specific IgE concentrations, and the number of neutrophils and eosinophils in the nasal mucosa were significantly increased at 2 h and decreased at 4 weeks after the last allergen challenge compared to controls. A total of 2119 DEG were identified, and their expression dynamics were clustered into 8 profiles. Enriched functions in Profile 5, which had a similar trend to clinical features, were mainly related to inflammatory and immune response to environmental factors, eosinophils and neutrophils chemotaxis, and cell migration. Gene co-expression Network for genes from profile 5 identified BCL3, NFKB2, SOCS3, and CD53 having a higher degree. Profile 6 showed persistence of inflammatory and immune response at 4 weeks after the last allergen challenge. Olfactory and coagulation functions were enriched mainly in profiles with downward trends.

**Conclusions:**

A wide range of genes with sequential cooperative action were identified to be associated with allergen exposure in AR. BCL3 may be the most vital in symptoms manifestation. Moreover, some inflammatory responses persisted for a period after allergen exposure, supporting a new treatment strategy of targeting inflammation out of season. This study may contribute to a better understanding of AR pathogenesis and provide potential therapeutic targets for AR patients.

**Supplementary Information:**

The online version contains supplementary material available at 10.1186/s12920-022-01389-4.

## Background

Allergic rhinitis (AR) is a common global health problem with increasing prevalence in China [1]; it is characterized by chronic inflammation of the nasal mucosa and symptoms of rhinorrhoea, nasal itching, obstruction, and sneezing associated with allergen exposure [2]. Although not life-threatening, AR status increases the risk for morbidity, adversely impacts quality of life, and has associated high costs. Management of AR consists of allergen avoidance, pharmacotherapy, and immunotherapy; however, current therapies are not satisfactory [3]. Thus, better treatment or prevention for AR requires a better understanding of the underlying mechanisms.

Although many factors play a role in AR, allergen exposure is thought to have a critical role in its pathogenesis, which is probably an important contributor to clinical manifestations of the disease. Many studies have explored the underlying mechanisms associated with allergen exposure by comparing the changes of immunological responses in seasonal AR patients during and out of pollen seasons [4–7]. As a local disease, it is better to collect nasal samples than peripheral blood samples. However, it is a challenge to collect intact nasal mucosa samples from AR patients due to its invasive properties, and thus nasal lavages [8] or nasal brushings [4, 5] are often sampled instead because they are non- or minimal invasive. The nasal brushing samples, while most representative of nasal mucosa, still may not fully reflect the conditions of whole nasal mucosa due to them being predominantly epithelial cells [9]. Furthermore, most previous studies were at individual component level rather than at high-throughput systematic level. Thus, in order to gain a comprehensive view of the molecular mechanisms in AR, we developed an OVA-induced AR mouse model and used a microarray method to systematically analyze dynamic gene expression changes in nasal mucosa at 2 h and 4 weeks after the last challenge, allowing it to mimic clinically “in season” and “out of season” phases, respectively.

## Materials and methods

### Animals

Male 6- to 8-wk-old BALB/c mice were obtained from Experimental Animal Centre (Beijing, China). Performance of all experimental procedures was done with approval from the Animal Care and Use Committee of Capital Medical University.

### AR mouse model

BALB/c mice were randomly assigned to three groups of six mice: control, OVA, and 4w-after group. Mice in control and OVA groups were sensitized and challenged with saline or ovalbumin (OVA), respectively, and euthanized 2 h after the last challenge. Mice in 4w-after group were sensitized and challenged with OVA and euthanized 4 weeks after the last challenge.

Animals were sensitized by intraperitoneally (i.p.) injecting saline or 50 μg OVA (grade V; Sigma, St Louis, Mo, USA) emulsified in 5 mg Al(OH)_3_ on days 1 and 7. From day 14, the animals were challenged by nasal instillation of saline or 50 μg OVA in 20 μL of 0.9% saline three successive days a week for three consecutive weeks.

At 2 h or 4 weeks after the last challenge, the blood was harvested under anesthesia with pentobarbital sodium (80 mg/kg, i.p.) and nasal mucosa was harvested after euthanizing by cervical dislocation. Nasal mucosa samples were used for RNA isolation and histological analysis, while serum was stored at − 70 °C and used for OVA-specific IgE detection.

### Measurement of nasal symptoms

The numbers of nasal rubbings and sneezes were counted for 20 min before the animals were euthanized, by three observers blinded to the experimental treatments given to the mice.

### Measurement of serum OVA-specific IgE

Serum OVA-specific IgE levels were determined by enzyme-linked immunosorbent assay (ELISA) as previously described [10]. Plates were coated with OVA and horseradish peroxidase-conjugated anti-mouse IgE (1:4,000) (Southern Biotech) was used. A standard curve was prepared from serial dilutions of an arbitrary standard, and the levels of OVA-specific IgE were expressed as arbitrary units (AU).

### Histological analysis

Nasal samples were embedded in paraffin and cut into 5 µm thick sections. The sample sections were stained with hematoxylin and eosin (H&E) to assess eosinophils. The sample sections were also assessed by means of immunohistochemistry staining for NIMP-R14 (Abcam, 2557, neutrophil marker), BCL3 (Santa Cruz, sc-185), NFKB2 ((Proteintech, 10409-2-AP), SOCS3 (Abcam, 280884), CD14 (HuaBio, ET1610-85) and TLR4 (HuaBio, ER1706-43). Horseradish peroxidase-conjugated secondary antibodies (Zhongshanjinqiao, Beijing, China) and substrate 3, 3′-diaminobenzidine were used, which rendered positive staining cells brown. The numbers of eosinophils, neutrophils, and positive staining cells for BCL3, NFKB2, SOCS3, CD14 and TLR4 were counted at 400X magnification by two observers who were blinded to the treatment.

### Microarray

Total RNA was isolated from the nasal mucosa samples, three mice each group, using the RNeasy mini kit (Qiagen, Valencia, CA, USA). Total cRNA was generated using a WT Expression Kit (Ambion; Austin, TX, USA) and labeled using a GeneChipWT Terminal Labeling Kit (Affymetrix; Santa Clara, CA, USA). The labeled cRNA was hybridized to GeneChip Mouse Gene 1.0 ST arrays (Affymetrix, Santa Clara, CA, USA) at 45 °C for 16 h and at the end of hybridization, the arrays were washed using the Fluidics station 450, prior to being scanned with a GeneChip Scanner 3000 7G (Affymetrix). The images of all arrays were transformed into digital data using the Command Console Software 4.0 (Affymetrix), and the data for each array were normalized by RMA + DABG normalization using the expression console.

### Bioinformatics analysis

#### Differentially expressed genes (DEG)

The random-variance model (RVM) F-test was applied to filter the DEG across the three groups because this test can raise the degrees of freedom effectively in small sample size cases [11]. After analysis for statistical significance and false discovery rate (FDR) analysis, the differentially expressed genes were selected (both *P* value and FDR less than 0.05).

#### Pathway enrichment analysis

Pathway analysis was performed to determine the significant pathway of the differential genes according to KEGG [12–14], Biocarta, and Reatome. Fisher's exact test and χ2 tests were used to select significant pathways with a threshold of *P* < 0.05.

#### Series tests of cluster (STC)

We also used STC method to analyze the expression dynamics of DEG by STEM software. To define a set of model profiles independent of the data, the amount of change a gene could exhibit among the three groups was set as ‘one unit’; thus, a changed gene could go up either one unit, stay the same, or go down one unit. Since this method relies on correlation, ‘one unit’ may be defined differently for different genes. For the three groups, this strategy results in 8 distinct profiles, and we, therefore, used a set of 8 unique model profiles to represent any expression changes that might occur. The raw expression values of DEG were converted into log2 ratios, where the ratios were determined based on the expression of the first group. The value of the first group after transformation was thus always 0. Passing the data normalization, each differential gene expression was assigned to the model profile that most closely matched the gene's expression profile as determined by correlation coefficient analysis [15, 16]. Significant profiles of STC analysis were determined by Fisher's exact test, which have significantly more genes assigned under the true ordering of time points compared to the average number assigned to the model profile in the permutation runs. And a *P* value of less than 0.05 is considered significant.

#### STC-gene ontology (GO) analysis

GO analysis was applied to the genes in each profile to identify the main function of the genes having the same expression trend according to the Gene Ontology, the key functional classification of NCBI [17]. Fisher’s exact test and *X*^2^ test were used to classify the GO category, with the *P*-values of less than 0.05 considered statistically significant. Enrichment is related to the specificity of the function. The higher the enrichment, the more specific the corresponding function.

#### Gene co-expression network

The co-expression network was generated using the Matlab 7.1-java software. The gene adjacency matrix M between two genes, i and j, which reflected the correlations between genes, was computed and presented as the network map.

In this map, each node describes a gene, and the relation between them is represented with a line segment. The most elementary characteristic of a node is its degree (the number of genes interacting with it). The greater degree of a gene indicated a more central role of the gene within the network. The network’s hub is the most important central gene, affecting the structure of the whole network and other associated genes. Clustering coefficient calculates the density in this neighborhood, with a larger clustering coefficient indicating that the actor is coupled with a stronger degree of "clique-like" local neighborhoods.

### Real-time PCR validation

The 21 DEG with higher degree identified by gene co-expression analysis were validated for their expression by real-time PCR. The RNA samples were same as those used in the microarray (see Additional file 1 for Primers). The gene expression levels were quantified relative to the expression of GAPDH or β-actin by Comparative Ct (delta delta Ct) method.

### Statistical analysis

Statistical analyses were performed using the SPSS 11.0 (SPSS, Inc, Chicago, IL, USA). Normally distributed data were expressed as mean ± SD, and the significance of any difference between the treatment groups was assessed using an unpaired Student’s *t*-test. Values of *P* < 0.05 were considered to be statistically significant.

## Results

### Resolution of AR symptoms and inflammation at 4 weeks after cessation of allergen exposure

**Fig. 1:**
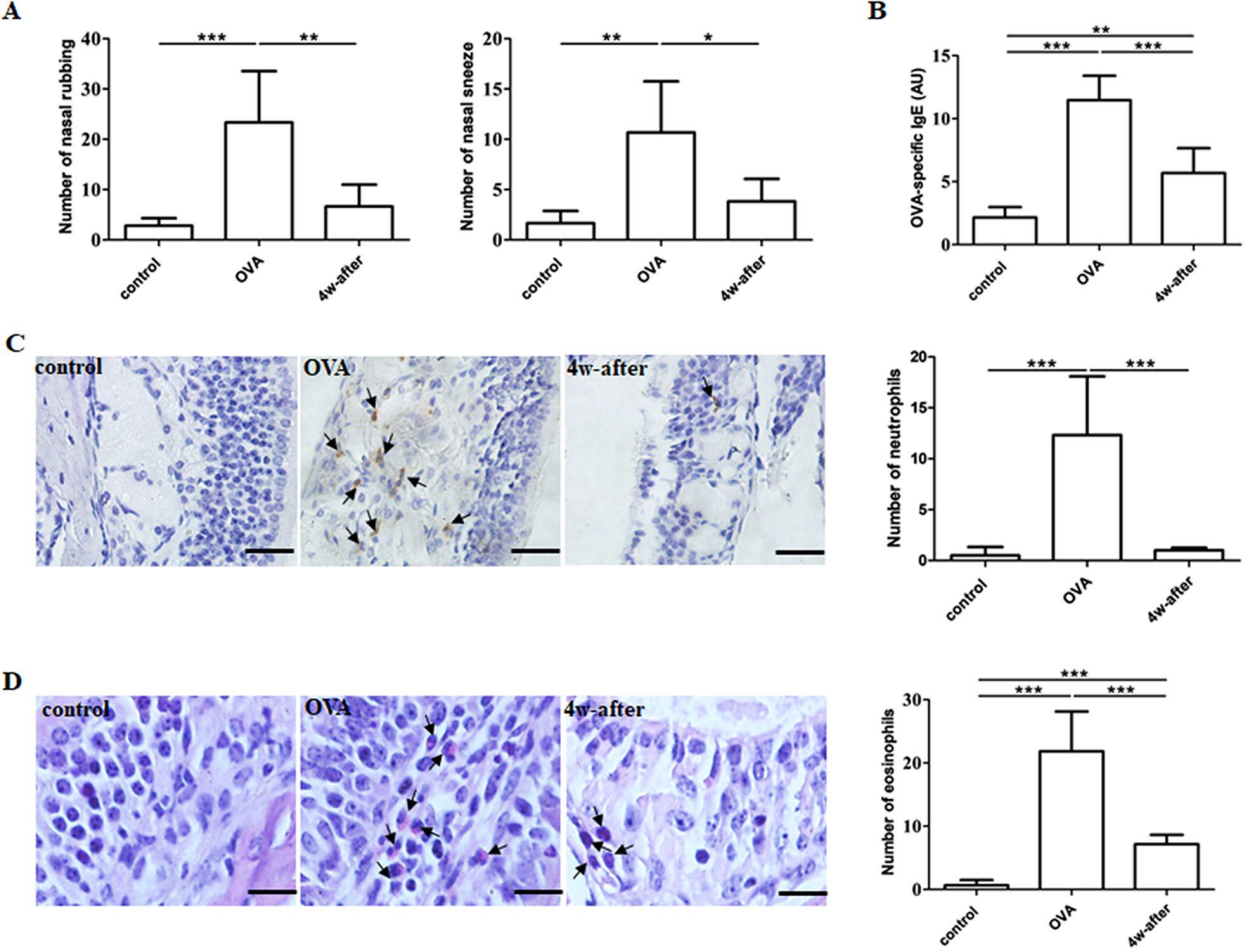
Effect of allergen exposure on allergic symptoms and immune responses in OVA-induced AR mice. **A** Nasal symptoms. **B** Serum OVA-specific IgE. **C**, **D** Representative images of neutrophils (**C**) and eosinophils (**D**) infiltrations in nasal mucosa and quantitative analysis (magnification, 400X); positive cells are indicated by arrows and scale bars are 20 μm for neutrophils and 10 μm for eosinophils. Control: sensitized and challenged with saline and euthanized 2 h after the last challenge; OVA: sensitized and challenged with OVA and euthanized 2 h after the last challenge; 4w-after: sensitized and challenged with OVA and euthanized 4w-after the last challenge. Data are expressed as the mean ± SD (n = 6 in each group). Results are representative of two independent experiments with similar results. *P < 0.05; **P < 0.01; ***P < 0.001

The number of nasal rubbings or sneezes, the concentration of serum OVA-specific IgE, and the number of neutrophils and eosinophils in the nasal mucosa were significantly higher in the OVA group compared to either the control group or the 4w-after group. Moreover, the OVA-specific IgE concentration and the number of eosinophils in the 4w-after group were higher than those in the control group (Fig. 1).

### DEG related to allergen exposure

To have a comprehensive understanding of the molecular mechanisms of AR, we performed a genome-wide transcriptional analysis of the nasal mucosal samples from the control, OVA, and 4w-after group mice. Overall, 2119 genes were differentially expressed across these three groups (see Additional file 2). The gene expression value per group was the geometric mean of the Robust Multichip Average (RMA) normalized gene signals of 3 samples per group.

### Pathway analysis of DEG

**Fig. 2:**
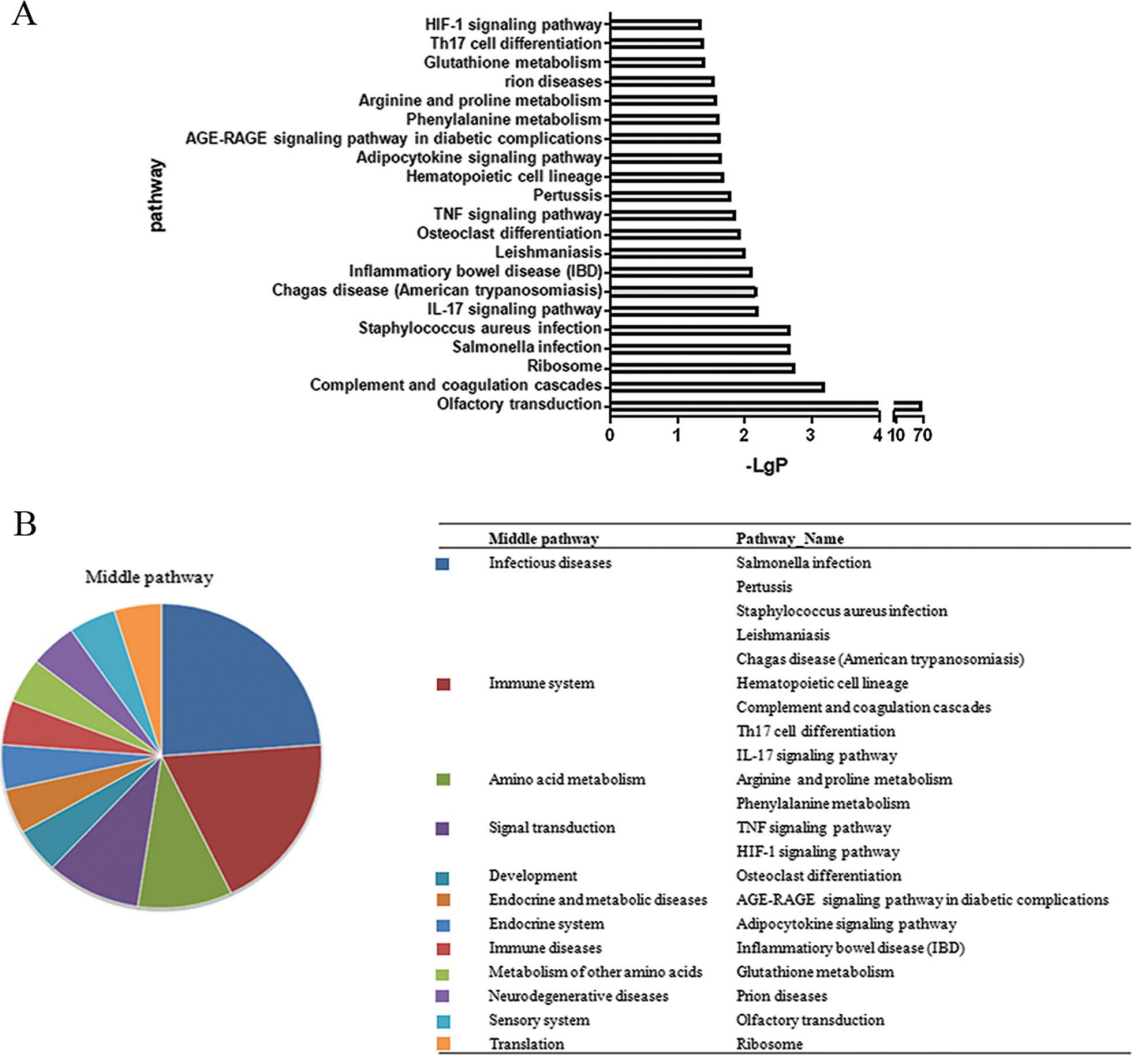
Pathway analysis of DEG identified in the three groups. **A** The significant pathway enriched. **B** The corresponding middle pathways of all significant pathways and their percentages

From 2119 DEG associated with allergen exposure, 21 enriched pathways with a *p*-value less than 0.05 were detected based on the KEGG database, with olfactory transduction being the most significant (Fig. 2A). The 21 pathways are mostly involved in infectious disease (salmonella, pertussis, staphylococcus aureus, leishmaniasis, and chagas disease), immune system (IL-17 signaling, Th17 cell differentiation, complement and coagulation cascades, and hematopoietic cell lineage), amino acid metabolism (arginine, proline and phenylalanine metabolism), and signal transduction (TNF signaling and HIF-1 signaling) (Fig. 2B). The genes corresponding to the 21 significant pathways are shown in Additional file 3.

### STC-GO analysis

**Fig. 3:**
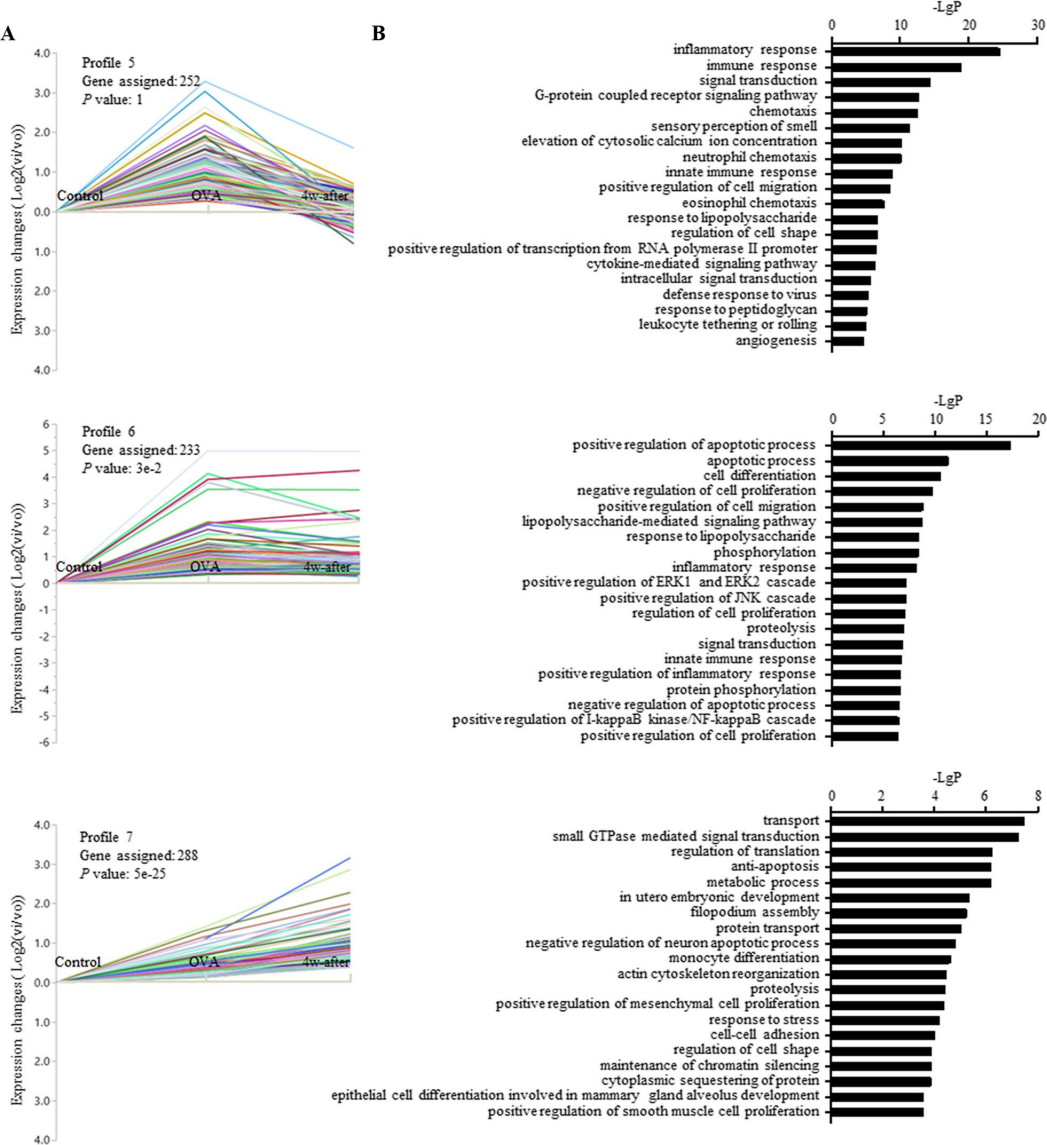
Profiles with upward gene expression in the OVA group compared to the control group and the top 20 functional GO categories within *P* < 0.01 in each profile

**Fig. 4:**
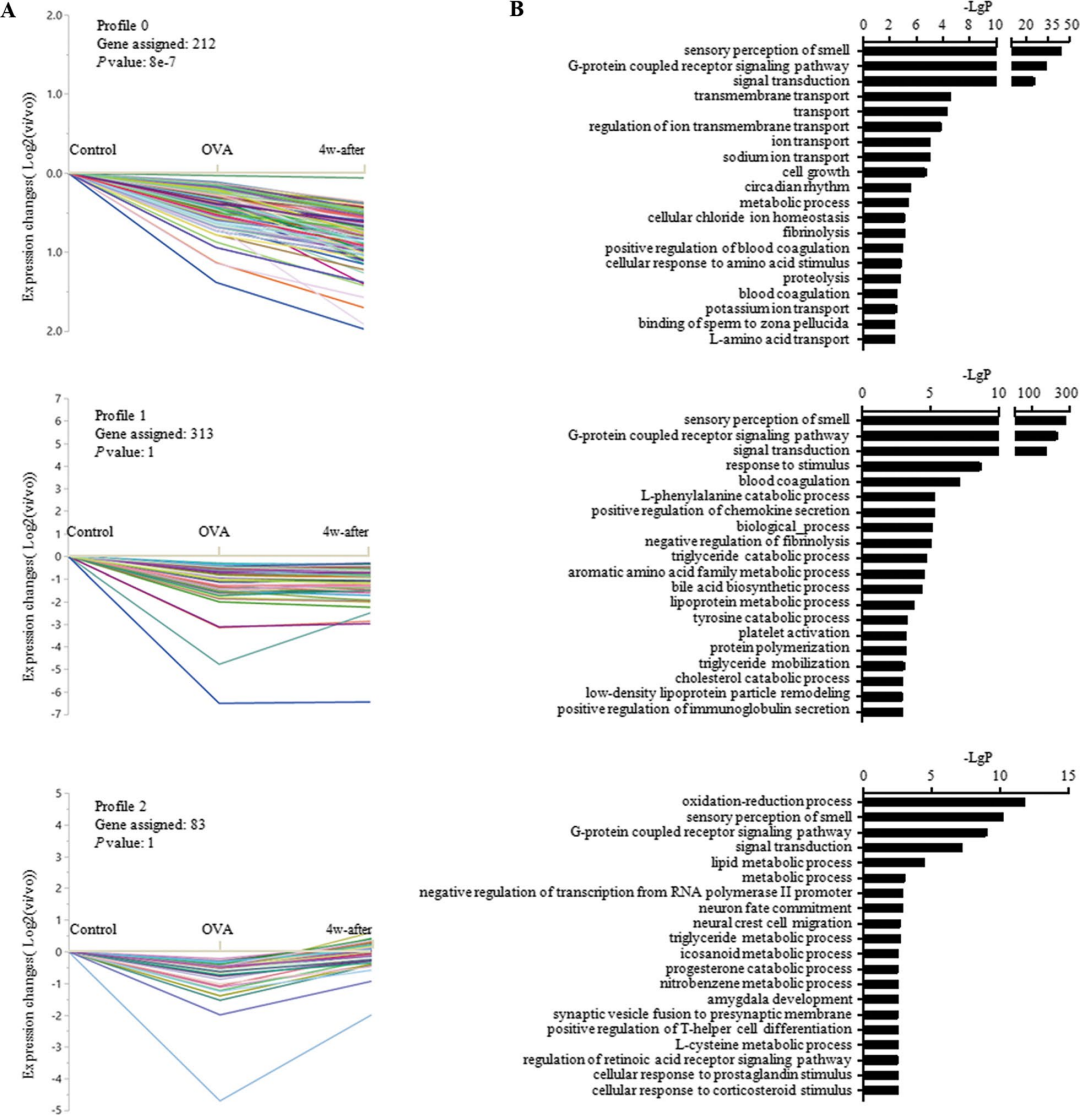
Profiles with downward gene expression in the OVA group compared to the control group and the top 20 functional GO categories within *P* < 0.01 in each profile

**Fig. 5:**
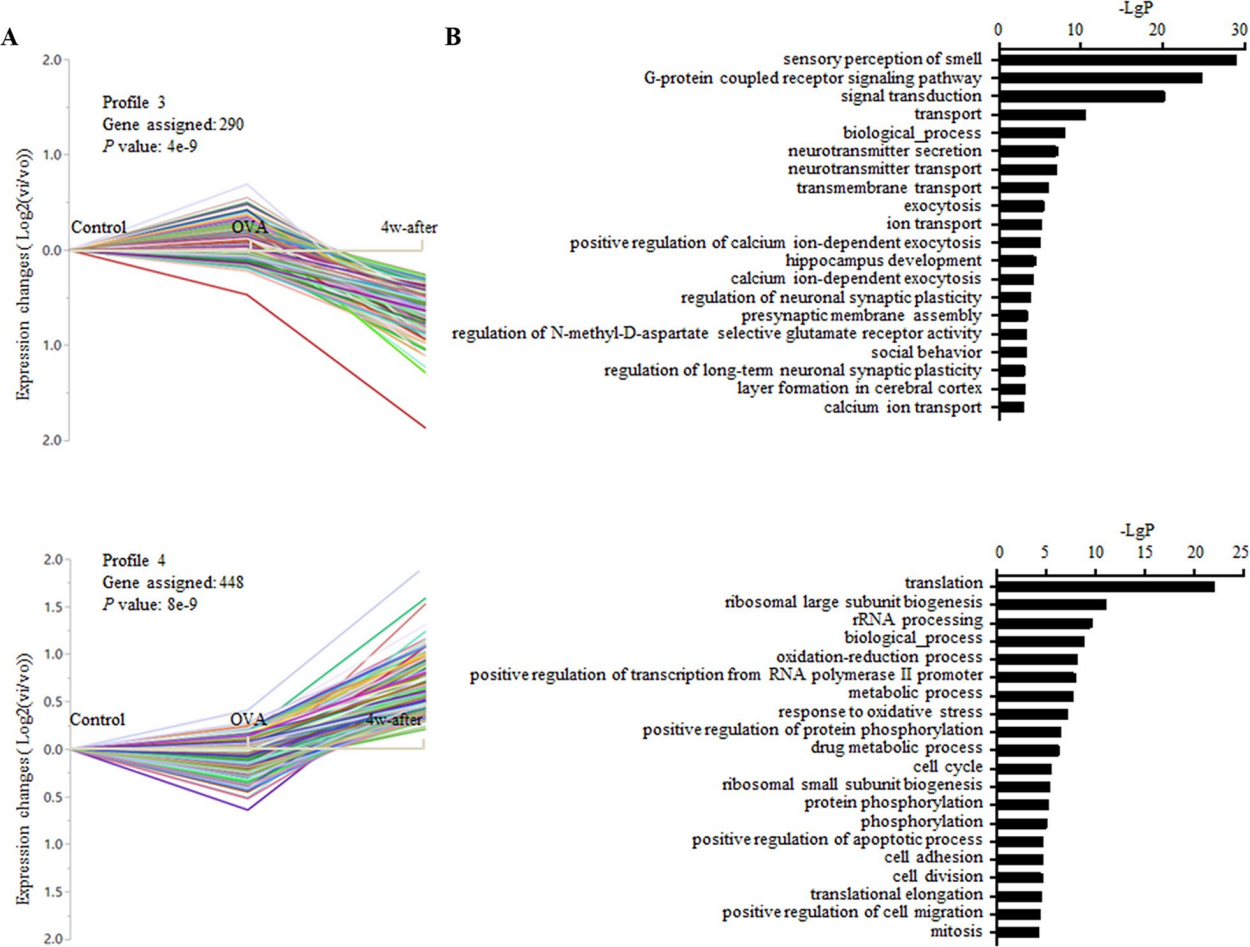
Profiles with constant gene expression in the OVA group compared to the control group and the top 20 functional GO categories within *P* < 0.01 in each profile

Eight model profiles were generated by STC analysis. Compared to the control group, the OVA group showed 3 profiles (5, 6, and 7) with an upward trend of gene expression (Fig. 3A), 3 profiles (0, 1, 2) with a downward trend (Fig. 4A), and 2 profiles (3, 4) unchanged (Fig. 5A). Four model profiles, 0, 3, 4, and 7, were identified with significance. The top 20 significant GO categories corresponding to each profile are shown in Figs. 3B, 4B, and 5B, respectively, with the genes corresponding to each GO included in Additional file 4.

DEG in profile 5 showed an upward trend in the OVA compared to control, while a downward trend is seen in the 4w-after compared to OVA. The most prominent functions enriched in profile 5 include inflammatory response, immune response, innate immune response, neutrophil chemotaxis, eosinophil chemotaxis, response to lipopolysaccharide, cytokine-mediated signaling, defense response to virus, positive regulation of cell migration, regulation of cell shape, and leukocyte tethering or rolling, which are mainly involved in regulating inflammatory and immune responses to environmental factors, eosinophils and neutrophils chemotaxis, and cell migration.

Main functions involved in profile 6, which showed an upward trend in the OVA compared to control, while being sustained in the 4w-after, consist of inflammatory response, innate immune response, positive regulation of inflammatory response, lipopolysaccharide-mediated signaling, response to lipopolysaccharide, and positive regulation of cell migration.

The enriched GO terms in profile 7 showed upregulation of genes in the OVA compared to control and 4w-after compared to OVA consisted of positive regulation of mesenchymal cell proliferation, positive regulation of smooth muscle cell proliferation, epithelial cell differentiation involved in mammary gland alveolus development, monocyte differentiation and cell–cell adhesion. Profile 4 with OVA sustained and 4w-after being upregulated consisted of enriched GO terms of translation, ribosomal large/small subunit biogenesis, cell cycle, mitosis, translation elongation, cell division, cell adhesion, positive regulation of cell migration, positive regulation of transcription from RNA polymerase II promoter, rRNA processing, which are involved in tissue repair.

The function of sensory perception of smell was enriched mainly in profiles with downward gene expression in the OVA group compared to the control group (54 genes in profile 0, 206 genes in profile 1, and 15 genes in profile 2, 47 genes in profile 3, and 26 genes in profile 5). Furthermore, blood coagulation was enriched in two profiles with downward trends, profiles 0 and 1. These two enriched functions identified by GO analysis were consistent with pathway analysis.

### Gene co-expression network for genes from profile 5 and 2

**Fig. 6:**
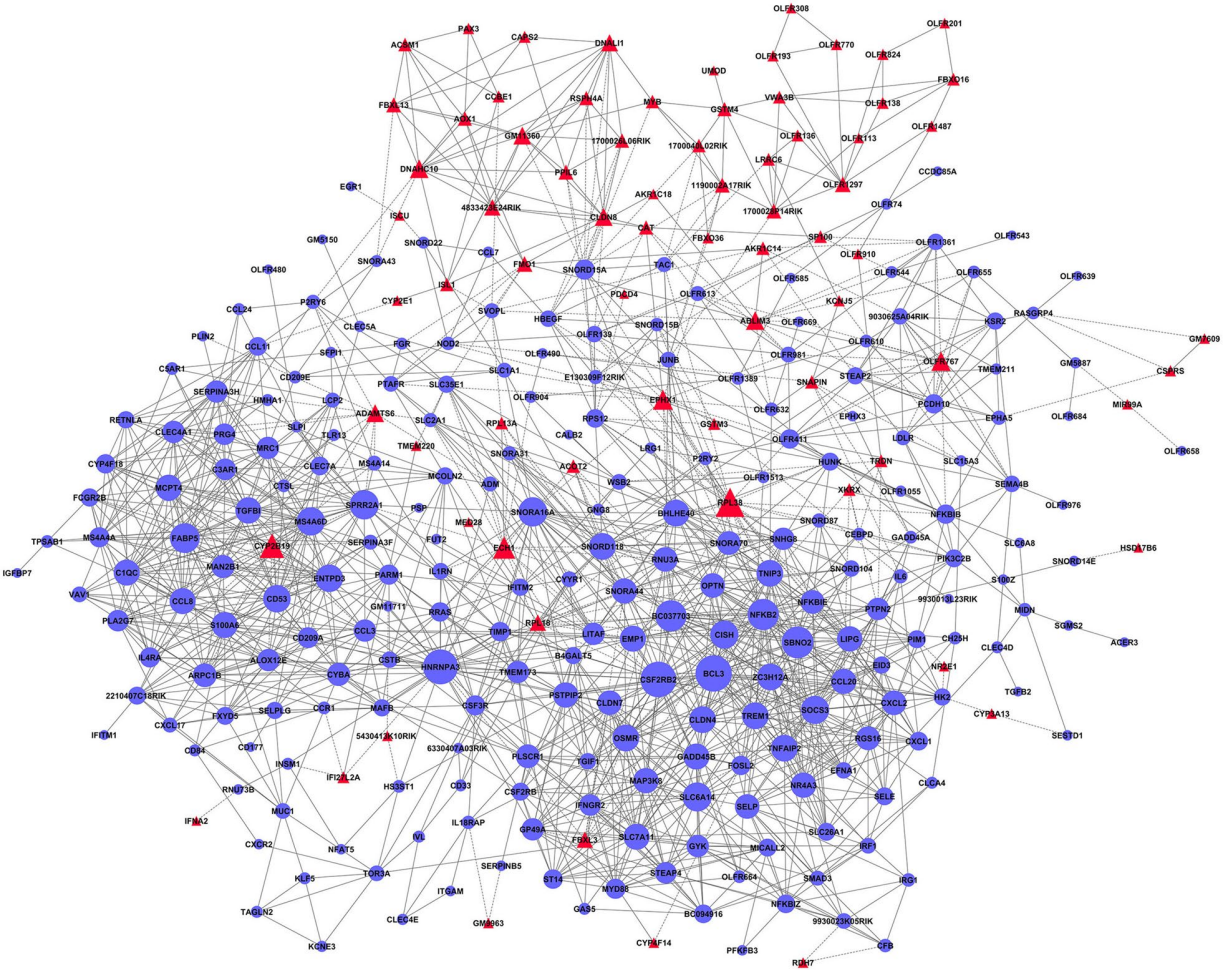
Gene co-expression network for genes in profile 5 and 2. Blue circles and red triangles represent genes from profile 5 and 2, respectively. Node represents gene and the size displays the degree (the number of genes interacting with it). Lines between two nodes indicate interactions between genes

To investigate which genes might play a pivotal role in controlling the clinical features of AR, gene co-expression network for profiles 5 and 2 that have similar and opposite trends to clinical features, respectively, was constructed to determine gene interactions (Fig. 6). Table 1 shows the top 21 genes with higher degree, all from profile 5 except RPL38. These genes include BCL3, NFKB2, SOCS3, CD53, and OSMR, with BCL3 having the highest degree.

**Table 1:** Top 21 genes with higher degrees identified by gene co-expression network analysis

Gene	Description	Clustering coefficient	Degree	k-core
BCL3	B cell leukemia/lymphoma 3	0.37687688	37	11
CSF2RB2	colony stimulating factor 2 receptor, beta 2, low-affinity (granulocyte–macrophage)	0.33492063	36	11
HNRNPA3	heterogeneous nuclear ribonucleoprotein A3	0.19663866	35	11
SBNO2	strawberry notch homolog 2 (Drosophila)	0.43225806	31	11
NFKB2	nuclear factor of kappa light polypeptide gene enhancer in B cells 2, p49/p100	0.43678161	30	11
FABP5	fatty acid binding protein 5, epidermal	0.47883598	28	11
RPL38	ribosomal protein L38	0.3048433	27	11
SLC6A14	solute carrier family 6 (neurotransmitter transporter), member 14	0.44159544	27	11
SPRR2A1	small proline-rich protein 2A1	0.31339031	27	11
CISH	cytokine inducible SH2-containing protein	0.43384615	26	11
MS4A6D	membrane-spanning 4-domains, subfamily A, member 6D	0.51076923	26	11
SOCS3	suppressor of cytokine signaling 3	0.49538462	26	11
CD53	CD53 antigen	0.42333333	25	11
ENTPD3	ectonucleoside triphosphate diphosphohydrolase 3	0.52666667	25	11
BHLHE40	basic helix-loop-helix family, member e40	0.37681159	24	11
CLDN4	claudin 4	0.43478261	24	11
OSMR	oncostatin M receptor	0.40942029	24	11
TNFAIP2	tumor necrosis factor, alpha-induced protein 2	0.45289855	24	11
TNIP3	TNFAIP3 interacting protein 3	0.4673913	24	11
TREM1	triggering receptor expressed on myeloid cells 1	0.55434783	24	11
ZC3H12A	zinc finger CCCH type containing 12A	0.48188406	24	11

### Validation of microarray results for genes of interest by real-time PCR and immunohistochemistry staining

The top 21 genes with higher degrees identified by gene co-expression analysis were validated by real-time PCR. While a few genes (HNRNPA3, SPRR2A1, and CD53) were inconsistent between real-time PCR and the microarray analysis, the others were (Table 2).

**Table 2:** Validation of the top 21 DEG identified by gene co-expression analysis by real-time PCR

Genes	2^−△△ct^(control)	2^−△△ct^(OVA)	2^−△△ct^(4w-after)
BCL3	1.45 ± 0.8	9.43 ± 0.87**	1.77 ± 0.28##
CSF2RB2	2.32 ± 2.3	18.04 ± 8.73*	3.4 ± 1.76#
HNRNPA3	1.18 ± 0.46	0.58 ± 0.07	0.85 ± 0.06##
SBNO2	1.38 ± 0.45	2.93 ± 0.55*	0.74 ± 0.22##
NFKB2	0.65 ± 0.32	2.17 ± 0.69*	0.51 ± 0.06#
FABP5	0.99 ± 0.14	4.18 ± 1.89*	2.16 ± 2.28
SLC6A14	0.81 ± 0.17	1.66 ± 0.09**	0.65 ± 0.08##
SPRR2A1	2.26 ± 1.65	5.16 ± 4.28	6.09 ± 8.1
CISH	0.87 ± 0.14	3.71 ± 0.78**	1.16 ± 0.56#
MS4A6D	0.62 ± 0.33	2.75 ± 0.99*	0.84 ± 0.25#
SOCS3	2.91 ± 3.35	15.8 ± 5.74*	4.25 ± 2.72#
CD53	0.85 ± 0.17	2.17 ± 0.48*	1.08 ± 0.37#
ENTPD3	1.68 ± 1.1	5.12 ± 1.17*	2.82 ± 0.56#
BHLHE40	3.34 ± 2.31	9.27 ± 1.94*	4.2 ± 0.88#
CLDN4	1.17 ± 0.85	3.34 ± 1.03*	1.07 ± 0.63#
OSMR	1.38 ± 0.45	2.93 ± 0.55*	0.74 ± 0.22##
TNFAIP2	0.70 ± 0.47	3.83 ± 1.61*	0.87 ± 0.42#
TNIP3	2.35 ± 1.19	14.28 ± 2.83**	2.25 ± 1.89##
TREM1	0.65 ± 0.38	1.82 ± 0.43**	0.51 ± 0.16##
ZC3H12A	1.03 ± 0.27	9.34 ± 1.78**	1.68 ± 0.33##
RPL38	0.89 ± 0.13	0.58 ± 0.39	3.24 ± 0.58##

The unpaired Student's t test was used to analyze gene expression.* *P* < 0.05,** *P* < 0.01, compared to control group; # *P* < 0.05,## *P* < 0.01, compared to OVA group

**Fig. 7:**
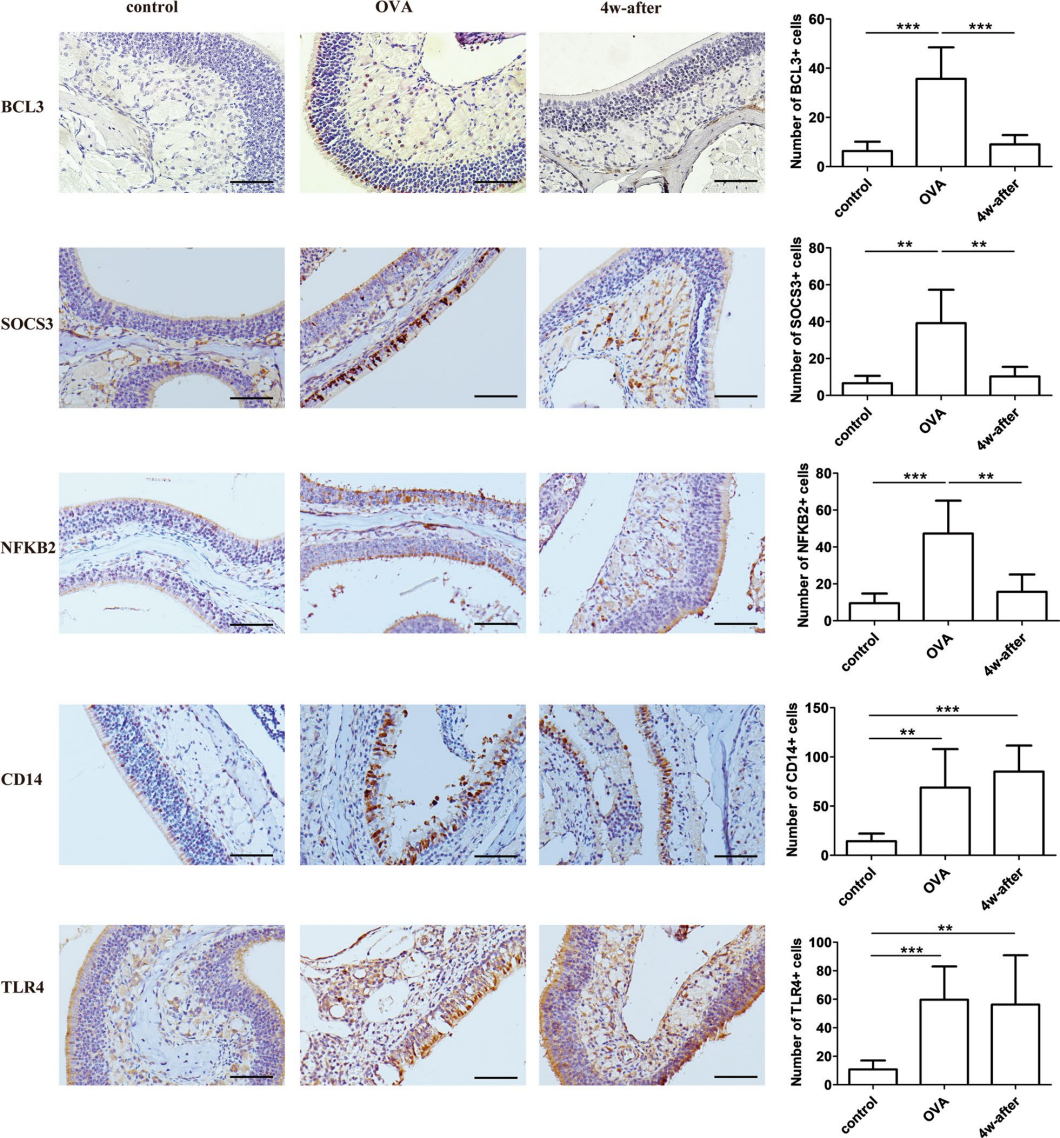
Expression BCL3, NFKB2, SOCS3, CD14 and TLR4 at the protein level by immunohistochemistry staining in nasal tissue. Representative images of staining and quantitative analysis (magnification, 200X); scale bars are 40 μm. Control: sensitized and challenged with saline and euthanized 2 h after the last challenge; OVA: sensitized and challenged with OVA and euthanized 2 h after the last challenge; 4w-after: sensitized and challenged with OVA and euthanized 4w-after the last challenge. Data are expressed as the mean ± SD (n = 6 in each group). Results are representative of two independent experiments with similar results. **P < 0.01; ***P < 0.001

Expression of BCL3, NFKB2, SOCS3, CD14 and TLR4 at the protein level were further validated by immunohistochemistry staining. The results were consistent with the microarray analysis (Fig. 7).

## Discussion

The typical clinical manifestations of AR are known to be associated with allergen exposure; however, the mechanisms underlying it are not fully understood. In this genomic study, we identified 2119 DEG by observing the dynamic gene expression changes in nasal tissue at 2 h and 4 weeks after the last challenge in an AR mouse model, which is similar to the natural course of in and out of season for seasonal AR patients. Many of these DEG in our mouse model such as TLR4, HMGB1, SOCS3, FETUB, FOS, JUN, CD14, VCAM1, IL-6, and IL-1β, have also been reported to be up-regulated in AR patients [18–24]; and 10 (AMD1, CDH26, CPA3, CYP2E1, FETUB, GCNT3, LDHA, POSTN, SLC6A14 and TPSAB1) of the 61 most discriminant epithelial genes between children with dust mite allergic rhinitis and healthy children identified in one previous study [25] were also found to be differentially expressed in our mouse model, supporting the results of this study. Using STC-GO and gene co-expression Network analyses, we identified several novel genes that have the potential to be used as therapeutic targets for AR patients.

Gene expression trend in Profile 5 perfectly matched the changes of typical nasal symptoms, which developed in the OVA group and resolved in 4w-after group, indicating the genes in this profile may have a prominent role in the development of clinical AR symptoms. Go terms analysis revealed these genes are involved in regulating inflammatory and innate immune response, the response to environmental factors, cell migration, leukocyte tethering or rolling, cell shape, and eosinophil and neutrophil chemotaxis. These findings are in accordance with the well-documented findings that AR is the result of allergen-triggered responses mediated by a series of inflammatory cells and mediators and the migration of inflammatory cells to the nasal mucosa [26]. Further co-expression network analysis identified the 21 most important genes associated with AR symptoms development, including BCL3, NFKB2, CD53, and SOCS3. BCL3, identified as the most important gene, belongs to the IkB (inhibitor of κB) proteins family and can either activate or repress gene transcription by forming complexes with two members of Nuclear Factor (NF)-kB family, p50 or p52 (NFKB2) homodimers [27]. BCL3 and NFKB2 are found to regulate germinal center formation and differentiation of follicular dendritic cells (FDCs) [27], which enhance memory B cell differentiation [28]. Moreover, one study using BCL3-deficient cells revealed it has an important role in regulating Th2 cell differentiation in vitro [29], which is a major immunologic response in AR. Therefore, future studies investigating the role of BCL3 in AR are warranted. Suppressor of cytokine signaling 3 (SOCS3) is predominantly expressed in Th2 cells and is upregulated in AR patients [16]. SOCS3 has an important role in regulating Th2 responses in allergic disease, and inhibiting its expression alleviates allergic inflammation [30, 31]. In line with this, our present study supports the importance of SOCS3 in AR by showing its association with AR symptoms manifestation. Another gene, CD53, a member of the tetraspanin superfamily, is expressed exclusively in immune cells. A previous study showed that the absence of CD53 causes pronounced immune dysfunction associated with adhesion-related migration defects [32], indicating it might affect immune responses in AR by regulating cell trafficking.

Contrary to their symptoms, AR patients show persistent inflammatory responses out of season with minimal neutrophil and eosinophil infiltration and ICAM-1 expression in nasal scraping samples [5] and elevated IL-18 and IL-1β in nasal secretions [24]. Based on these findings, treatments targeting inflammation when symptoms are absent have been proposed. Also, prophylactic treatment with a combination of anti-leukotrienes and antihistamine or anti-inflammation treatments before pollen season has been applied in clinical practice and is proven to reduce nasal symptoms [33, 34]. In this present study, GO terms of immune response, inflammatory response, response to lipopolysaccharide were exclusively within top 20 GO terms of profile 5 and 6, and those in the latter had elevated inflammation in mice with symptoms absent at 4 weeks after allergen exposure, which supports the existence of persistent inflammation in AR patient out of season or without symptoms. Of these genes, IL-1β confirmed a previous study, which found the elevated levels in nasal secretions of AR patients out of season [24]. Furthermore, TLR4 and CD14 attracted our attention because the latter is a pattern recognition receptor and mainly acts as a co-receptor with TLR4 to facilitate responses to low doses of lipopolysaccharide [35]. The up-regulation of these two genes in AR has already been reported [18, 22]. Moreover, both agonist and antagonist of TLR4 are used to modify AR or asthma based on activation of TLR4 acting as an adjuvant in allergic vaccines to induce tolerance [36, 37]. Our findings of the persistence of TLR4 and CD14 in mice after allergen exposure further suggest inflammation persists out of season. Therefore, IL-1β, TLR4, and CD14 could be targeted for out-of-season treatment in AR.

Patients with AR often suffer from olfactory dysfunction [38]. Consistent with our results, it is the most significantly disregulated pathway. Furthermore, STC-GO analysis showed that sensory perception of smell was enriched in the profiles with downward trends. Given the lack of smell function testing in this study, it will require further investigation to see if a decrease in expression of these genes is related to olfactory dysfunction. One recent study showed that olfactory sphere cells are significantly reduced in the AR mouse model, and TNFα combined with IL-5 had an apoptotic effect on these cells [39]. Therefore, the impact of diverse inflammatory factors on these genes needs to be further investigated. Most (307) of the down-regulated olfactory genes are still decreased at 4 weeks after allergen exposure and only few (41) return to normal, indicating the cessation of allergen exposure for 4 weeks may not be long enough to restore the olfactory function. This is consistent with a recent study that showed olfactory dysfunction in out-of-season patients with seasonal AR [40]. This evidence suggests that remaining inflammation may affect this progress.

The change in coagulation function has been investigated in asthma, and most studies have shown it is activated [41]. However, few studies have been done on it in AR. One study showed that the F2 and F10 genes were increased in nasal mucosa of AR mice [42]. Another proteomic study found that 6 of 10 coagulation-related proteins (F12, F9, PLG, F2, TF, and A2M) were up-regulated and 4 (F5, F11, SERPINF2, CPB2) down-regulated in AR patient serum [43]. While, in the present study, we found coagulation function (F2, F12 F9, HRG, FGG, serpinC1, PLG, and FGB) was down-regulated in nasal mucosa of AR mice, it did not return to normal after 4 weeks cessation of allergen exposure. This is consistent with the previous finding of delayed formation of thrombin in atopic patients [44]. These conflicting results indicate that more studies are needed to clarify the function of coagulation in AR.

The present study has some limitations. First, the gene expression microarray analysis shows altered transcription of genes, and the corresponding protein changes may not correspond. Second, the sensitivity of the microarray analysis may not be high enough to detect low-copy-number transcripts; thus, low-expression genes may not be accurate. Third, bioinformatics analysis is limited by our knowledge of known murine genes, their functions, and their relations. Fourth, the sample size is relatively small and future study with more samples should be done. Finally, the AR model used here is induced by OVA rather than clinically relevant pollen or house dust mite.

## Conclusions

A wide range of genes with sequential cooperative action were identified to be associated with allergen exposure in AR. Several genes, such as BCL3, NFKB2, CD53, and SOCS3, might play vital roles in AR manifestations. Moreover, the persistent inflammatory responses after allergen exposure support the proposed new treatment strategy of targeting inflammation out of season. This study may improve the understanding of AR molecular mechanisms and provide potential therapeutic targets for AR patients.

## Supplementary Information

**Additional file 1: Table S1**. Primers used for qRT-PCR.

**Additional file 2: Table S2**. Differentially expressed genes.

**Additional file 3: Table S3**. The genes corresponding to the 21 significant pathways.

**Additional file 4: Table S4**. Genes corresponding to top 20 significant GO categories in 8 profiles.

## Data Availability

The datasets generated and/or analysed during the current study are available in NCBI Gene Expression Omnibus, https://www.ncbi.nlm.nih.gov/geo, GEO. Submission: GSE52804.

## Abbreviations

AR: Allergic rhinitis
OVA: Ovalbumin
AU: Arbitrary units
DEG: Differentially expressed genes
RVM: Random-variance model
STC: Series tests of cluster
GO: Gene Ontology
